# Weight estimation in people aged 65 years and over admitted to hospitalisations units

**DOI:** 10.3389/fnut.2026.1756595

**Published:** 2026-03-04

**Authors:** Joaquín Moncho, Eva María Trescastro-López, Patricia Navarro-Llopes, Cristina Carretero-Randez, Rafaela Camacho-Bejarano, María Trinidad Castillo-García, María Isabel Orts-Cortés, NUTRIFAG Group

**Affiliations:** 1 Research Unit for the Analysis of Mortality and Health Statistics, Department of Community Nursing, Preventive Medicine, Public Health and History of Science, University of Alicante, Alicante, Spain; 2 Alicante Institute for Health and Biomedical Research (Group 23, ISABIAL), Alicante, Spain; 3 Department of Nursing, University of Alicante, Alicante, Spain; 4 Balmis Research Group in History of Science, Health Care and Food, University of Alicante, Alicante, Spain; 5 Research Group on Applied Dietetics, Nutrition and Body Composition, University of Alicante, Alicante, Spain; 6 Faculty of Health Sciences, University of Alicante, Alicante, Spain; 7 Nursing in the Burns Unit, University and Polytechnic Hospital La Fe, Valencia, Spain; 8 Doctorate in Health Sciences, Community Health and History of Science Team, Nursing Department, University of Alicante, Alicante, Spain; 9 Nursing Research Group: Infection, Inflammation and Chronicity (IIS La Fe), Hospital Universitario y Politécnico La Fe, Valencia, Spain; 10 Department of Nursing, Faculty of Nursing, University of Huelva, Huelva, Spain; 11 Nursing and Healthcare Research Unit (Investén-ISCIII), Instituto de Salud Carlos III, Madrid, Spain; 12 Research Network on Chronicity, Primary Care and Health Promotion (RICAPPS), Madrid, Spain; 13 Dr. Balmis General University Hospital, Alicante Institute for Health and Biomedical Research (Group 20, ISABIAL), Alicante, Spain; 14 CIBER of Frailty and Healthy Ageing (CIBERFES), Instituto de Salud Carlos III, Madrid, Spain

**Keywords:** aged, anthropometry, body weight, hospitalization, malnutrition, nutrition

## Abstract

**Introduction:**

Malnutrition remains highly prevalent among hospitalised older adults, yet obtaining an accurate body weight, an essential parameter for nutritional assessment, medication dosing, and broader clinical decision-making, often poses substantial challenges in routine care. In patients with reduced mobility, direct weight measurement is frequently unfeasible due to limitations in available equipment and clinical resources. This study aimed to evaluate the validity and accuracy of three commonly used body-weight estimation equations and to develop and internally validate a new predictive model specifically designed for hospitalised adults aged ≥65 years.

**Materials and methods:**

A multicentre, cross-sectional study was conducted in nine Spanish hospitals using data from the NUTRIFAG research project, which investigates the nutritional status of hospitalized older adults and was carried out between October 2022 and February 2024. Measured weight, mid-arm circumference (BC), mid-calf circumference (CC), and heel–knee length (KH) were obtained following standardized procedures. Agreement between measured and estimated weight from three published equations was analyzed using intraclass correlation coefficients (ICC), linear regression, and Bland–Altman plots. A new predictive equation was developed through multiple linear regression in a derivation sample (60%) and validated in an independent subsample (40%). The impact of weight estimation on BMI and BMI-based nutritional according to GLIM criteria was also examined.

**Results:**

A total of 1,196 patients were included (54.7% men, 45.3% women; mean age 78.27 ± 7.95 years). All three reference equations systematically underestimated measured weight, particularly in men. Equation 1 yielded the highest consistency ICC (men = 0.769; women = 0.834) but lower absolute agreement. The new two-variable model (BC + CC) demonstrated good predictive capacity (R^2^ = 0.639 in men; R^2^ = 0.704 in women) and minimal mean bias (< 1 kg). In the validation sample, ICC was 0.764 (95% CI 0.710–0.810) for men and 0.824 (95% CI 0.777–0.862) for women, confirming excellent reliability without systematic bias. Previously published equations markedly underestimated BMI, resulting in a substantial overestimation of low BMI prevalence according to GLIM criteria, whereas the new equation showed minimal bias and preserved BMI classification comparable to measured weight.

**Conclusion:**

The proposed equation based on mid-arm and mid-calf circumferences offers a simple, accurate, and clinically applicable method for estimating body weight in hospitalized older adults with reduced mobility. By reducing BMI classification errors, its use may contribute to more appropriate nutritional assessment and clinical decision-making. External validation in other settings is recommended.

## 1 Introduction

Malnutrition in hospitalized patients, particularly in adults aged 65 years and older, represents a frequent yet often underdiagnosed clinical condition, with a reported prevalence ranging from 20 to 50% (1–2). This condition is associated with relevant clinical consequences, including an increased risk of complications—such as nosocomial infections—longer hospital stays, and higher mortality rates. These adverse outcomes underscore the importance of early nutritional assessment and timely nutritional interventions (3–5).

Accurate estimation of body weight is of critical clinical relevance, as it is essential for medication dosing, body mass index (BMI) calculation, and nutritional status assessment (5–6). However, in hospitalized patients—particularly older adults with functional limitations—direct weight measurement using conventional scales may be unfeasible due to mobility restrictions or the lack of appropriate equipment (3). In such cases, the use of anthropometric-based predictive equations offers a practical and feasible alternative in clinical practice (7–8).

Beyond anthropometric assessment, inaccuracies in body weight measurement can also have downstream effects on energy-related calculations and metabolic evaluation. Recent evidence comparing different methods for estimating basal metabolic rate has shown that discrepancies between measurement techniques may lead to clinically relevant differences in estimated energy requirements, with potential implications for nutritional planning and clinical decision-making (9). These findings highlight the broader clinical importance of precise weight- and body size–dependent measurements, particularly in metabolically vulnerable populations. In this context, improving the accuracy of body weight estimation becomes particularly relevant in hospitalised older adults, in whom direct measurement is frequently unfeasible.

Several predictive equations have been developed using variables such as knee height, arm, calf, or waist circumference, some of which are specifically designed for individuals over 60 years of age or bedridden patients (10). Nevertheless, many of these equations require complex measurements, trained personnel, or equipment not routinely available in hospital settings, limiting their applicability in patients with reduced mobility (7). Therefore, selecting predictive equations based on a limited number of simple, reproducible, and clinically accessible measurements is essential for use under real hospital conditions.

Implementing a more accurate and practical predictive equation could have a significant impact on patient care by improving the precision of nutritional assessment, reducing dosing errors, and supporting clinical decision-making when actual body weight cannot be obtained (5, 11).

The objective of this research is to evaluate the validity and accuracy of three commonly used body-weight estimation equations and to develop and internally validate a new predictive model specifically designed for hospitalised adults aged ≥65 years.

## 2 Materials and methods

### 2.1 Design and setting

A multicentre, observational, cross-sectional research was conducted from October 2022 to February 2024 within the NUTRIFAG study framework which focuses on the early detection and comprehensive management of malnutrition and dysphagia in hospitalized adults aged 65 years or older.

The study was carried out in the medical and surgical hospitalization units of nine public hospitals in Spain: General University Hospital of Alicante Dr. Balmis (HGUA), Gran Canaria Island University Hospital Complex (HUIGC), Lozano Blesa University Hospital of Zaragoza (HCULB), University Hospital of Gran Canaria Doctor Negrín, Las Palmas (HUGCN), Juan Ramón Jiménez University Hospital of Huelva (HUJRJ), Arnau de Vilanova University Hospital of Lleida (HUAV), Reina Sofía General University Hospital of Murcia (HGURS), University Hospital of Navarra (HUN), and University and Polytechnic Hospital La Fe, Valencia (HUPLF).

### 2.2 Participants

Participants were selected through consecutive sampling and included patients aged 65 years and older who were admitted to the medical and surgical inpatient units of the participating hospitals, had an expected hospital stay of at least 48 h, provided written informed consent (or through their legal guardian), and for whom both measured and estimated weight could be obtained.

Patients receiving enteral or parenteral nutrition, those with terminal illness or primary diagnosis of cancer, and those admitted to critical care units or with a diagnosis of COVID-19 at admission or during hospitalization were excluded.

The study sample included patients who met all predefined inclusion criteria and retained sufficient mobility to allow both direct and estimated weight measurements. This enabled a valid comparison of the performance and accuracy of the three weight-estimation equations across different functional profiles. This approach provided a solid basis for proposing an improved and optimised predictive equation that could be validated and applied specifically to hospitalised patients with limited mobility in future studies.

### 2.3 Variables and instruments

This study utilized data from the NUTRIFAG research project (12). The study variables were organized into three categories: socio-demographic, contextual, and anthropometric variables.

- Socio-demographic variables: age and sex (male or female).
- Contextual variables: participating hospital (nine centres).
- Anthropometric variables: measured weight (kg), measured height and proxy anthropometric measures used to estimate weight, including mid-arm circumference (BC), mid-calf circumference (CC), and heel–knee length (KH), depending on the equation applied.
- Assessment of the impact of weight estimation on body mass index (BMI): the difference between measured BMI and estimated BMI was calculated. For clinical interpretation, low BMI cut-off points proposed by the Global Leadership Initiative on Malnutrition (GLIM) (13) consensus were applied, using age-specific thresholds (<70 and ≥70 years). Agreement and changes in low BMI classification were analysed by comparing BMI calculated using measured weight with BMI derived from estimated weight, with analyses stratified by sex.

### 2.4 Equations used for weight estimation

This study evaluates three equations selected primarily for their simplicity and clinical applicability, particularly among patients with mobility impairments. These formulas were chosen not only for their operational feasibility in hospital environments but also because they were applied within the NUTRIFAG study framework (12).

Equation 1: Martín and Hernández (14) developed for predicting body weight in adults aged 20–50 years is detailed below.


WOMEN=[BCx1,854]+[CCx1,247]−33,770

MEN=[BCx1,773]+[CCx1,334]−33,474

Where BC = Mid-arm Circumference (cm); CC = Mid-Calf Circumference (cm).

Equation 2 Elia and British Association for Parenteral and Enteral Nutrition (BAPEN) (15) developed for Caucasian adults aged 60–80 years, estimating weight from Knee height and arm circumference.


WOMEN=[KHx1,09]+[BCx2,68]−65,51

MEN=[KHx1,10]+[BCx3,07]−75,81

Where KH = Knee Height (cm); BC = Mid-arm Circumference (cm).

Equation 3 Jung et al. (16) developed for Caucasian adults aged 60–80 years, using knee height and arm circumference.


WOMEN=[KHx1,01]+[BCx2,81]−66,04

MEN=[KHx1,10]+[BCx3,07]−75,81

where KH = Knee Height (cm); BC = Mid-arm Circumference (cm).

### 2.5 Data collection

Data collection and patient recruitment followed the procedures established by the NUTRIFAG research project (12). In each participating hospital, a multidisciplinary team comprising of nurses, nutritionists, and endocrinologists was established, led by a site coordinator. The coordinators were responsible for overseeing recruitment and data collection at their respective centres.

At each site, one investigator was responsible for the daily screening of hospital admission records and electronic medical charts to identify eligible patients. Eligible individuals were informed personally about the study, provided with an information sheet, and invited to participate. Written informed consent was obtained prior to inclusion.

Within the first 24–48 h of admission, nurses and/or nutritionists collected socio-demographic data (age, sex), contextual data (hospital), and anthropometric measures (measured weight, BC, CC, KH). Depending on each centre’s resources, anthropometric measurements were obtained by trained nursing or nutrition staff. All professionals involved in anthropometric data collection received standardized training prior to study initiation.

A detailed description of the measurement procedures and equipment used for each anthropometric variable is provided in Supplementary material.

Data were collected and managed using the REDCap (Research Electronic Data Capture) platform, hosted at the Institute for Biomedical Health Research of Alicante (ISABIAL) (17, 18). REDCap is a secure, web-based application that enables the creation of electronic case report forms (eCRFs) and supports both online and offline data entry via computers and mobile devices. The system includes scheduling and alert functions for data collection and monitoring. All data collectors were trained in the use of the REDCap platform prior to study commencement.

### 2.6 Data analysis

First, a descriptive analysis of the study variables was performed. Regarding the qualitative variables, absolute frequencies and percentages were calculated; for quantitative variables, measures of central tendency (mean and median) and dispersion (standard deviation and percentiles) were obtained.

For the analysis of concordance between the actual weight measurements and those estimated by each of the three equations considered in the study, intraclass correlation coefficients (ICC) were calculated —both the consistency (CCIC) and absolute agreement (CCIA) forms— disaggregated by sex, using a two-way mixed-effects model for each equation (14–16). The agreement analysis between measured and estimated weight was complemented with Bland–Altman plots.

In order to develop the new predictive body weight equation, the total sample was randomly divided into two independent subsamples. Sixty percent (60%) of participants were used as the training (derivation) sample to identify significant predictor variables through multiple linear regression analysis. Based on the coefficients obtained, a sex-specific predictive equation was formulated. The remaining 40% of participants constituted the validation subsample, used to assess predictive performance and agreement with measured weight using the intraclass correlation coefficient (ICC, absolute agreement type) and Bland–Altman plots.

This procedure allowed verification of the stability and accuracy of the model, minimized the risk of overfitting, and ensured its applicability to independent samples. To assess the impact of weight estimation on BMI and on BMI categorization according to GLIM criteria, mean measured and estimated BMI values were calculated for each equation, stratified by sex, together with the percentages of participants classified as low BMI according to GLIM cut-offs. Paired *t*-tests were used to analyse differences between measured and estimated BMI values, and McNemar’s test was applied to assess differences in low BMI classification, in both cases, the Bonferroni correction for multiple comparisons was applied. A significance level of *p* < 0.05 was established. All statistical analyses were conducted using IBM SPSS Statistics, version 29.0.

### 2.7 Ethical considerations

The project has been approved by the Ethics and Research Committee of the reference centre (No: 200026) and by the respective Ethics and Research Committees of the participating hospitals. The project has been designed based on current Spanish legislation on research, specifically Law 14/2007, of 3 July, on Biomedical Research and Law 3/2018, of 5 December, on the protection of personal data and guarantee of digital rights. On this basis, written informed consent was obtained from all study participants. The information was provided in writing and verbally and included the nature, significance, implications, and risks of the research, as well as their right to leave the study at any time they considered appropriate and without the need for specific justification. The information was adapted to persons with disabilities in accessible conditions and formats appropriate to their needs. If the research subject was unable to read or write, consent could be given by a legal representative or by any legally acceptable means that allowed the subject’s wishes to be recorded. In addition, personal data protection was ensured through a pseudonymisation process, and the information necessary to carry out this study was collected. The principles established in the Declaration of Helsinki were respected in the development of the study.

## 3 Results

A total of 1,196 patients were recruited, of whom 54.7% were male (*n* = 654) and 45.3% female (*n* = 542). The mean age of the sample was 78.27 ± 7.95 years (77.08 ± 7.67 in men and 78.89 ± 8.05 in women). Table 1 shows the distribution of patients by sex and hospital.

**Table 1:** Distribution of patients by sex and hospital.

Hospital	Total *n* (%)	Sex	
		Male *n* (%)	Female *n* (%)
HUJRJ (Huelva)^a^	128 (10.7)	67 (10.2)	61 (11.3)
HCULB (Zaragoza)^b^	90 (7.5)	57 (8.7)	33 (6.1)
HUIGC (Las Palmas)^c^	23 (1.9)	14 (1.2)	9 (0.8)
HUGCN (Las Palmas)^d^	120 (10.0)	56 (8.6)	64 (11.8)
HUAV (Lleida)^e^	261 (21.8)	161 (24.6)	100 (18.5)
HUN (Pamplona)^f^	143 (12.0)	67 (10.2)	76 (14.0)
HGUA (Alicante)^g^	154 (12.9)	85 (13.0)	69 (12.7)
HUPLF (Valencia)^h^	107 (8.9)	66 (10.1)	41 (7.6)
HGURS (Murcia)^i^	170 (14.2)	81 (12.4)	89 (16.4)
N total	1,196 (100.0)	654 (54.7)	542 (45.3)

^a^HUJRJ: Juan Ramón Jiménez University Hospital of Huelva; ^b^HCULB: Lozano Blesa University Hospital of Zaragoza; ^c^HUIGC: Gran Canaria Island University Hospital Complex; ^d^HUGCN: University Hospital Doctor Negrín; ^e^HUAV: Arnau de Vilanova University Hospital; ^f^HUN: University Hospital of Navarra; ^g^HGUA: General University Hospital Dr. Balmis; ^h^HUPLF: University and Polytechnic Hospital La Fe; ^i^HGURS: Reina Sofía General University Hospital.

Percentages refer to the proportion of the total sample (*N* = 1,196).

Table 2 presents the measured and estimated body weights obtained from the three predictive equations, stratified by sex. The mean measured weight was 76.26 ± 14.86 kg in men and 67.53 ± 15.53 kg in women. In both sexes, all three equations underestimated the measured weight. Among men, the mean estimated weight ranged from 62.84 ± 12.43 kg (Equation 1) to 65.52 ± 14.30 kg (Equations 2, 3). Among women, the corresponding values ranged from 60.61 ± 16.91 kg (Equation 3) to 61.06 ± 16.35 kg (Equation 2). Median and percentile values followed a similar pattern, with narrower ranges for estimated compared to measured weights. The lowest estimates were consistently obtained using Equation 1, in both sexes.

**Table 2:** Measured and estimated weights according to three predictive equations, stratified by sex.

Variable	Measured weight	Equation 1ᵃ	Equation 2ᵇ	Equation 3ᶜ
Male (*n* = 654)				
Mean ± SD	76.26 ± 14.86	62.84 ± 12.43	65.52 ± 14.30	65.52 ± 14.30
Median	75.50	62.11	64.19	64.19
Range	39.40–138.00	31.79–204.70	29.59–125.45	29.59–125.45
P10–P90	59.00–95.55	48.55–78.08	48.47–85.10	48.47–85.10
Female (*n* = 542)				
Mean ± SD	67.53 ± 15.53	60.99 ± 14.61	61.06 ± 16.35	60.61 ± 16.91
Median	65.90	59.91	59.73	59.67
Range	30.00–126.00	21.44–161.23	24.56–206.98	24.00–214.22
P10–P90	49.87–87.64	45.03–78.15	42.88–87.43	42.11–87.46

^a^Equation 1 (14); ^b^Equation 2 (15); ^c^Equation 3 (16).

All values are expressed in kilograms (kg); SD, standard deviation; P, percentile.

### 3.1 Agreement between measured and estimated body weight according to predictive equations

Table 3 shows the intraclass correlation coefficients (ICC) for both consistency and absolute agreement between measured and estimated body weight, stratified by sex. Among men, consistency values ranged from 0.736 to 0.769, whereas absolute agreement values were lower (0.498–0.581). Among women, ICCs were generally higher, with consistency ranging from 0.768 to 0.834 and absolute agreement from 0.702 to 0.765.

**Table 3:** Intraclass correlation coefficients (ICC) for consistency and absolute agreement according to predictive equation and sex.

Sex	Equation	Consistency (single measures)	95% CI	Absolute agreement (single measures)	95% CI
Male	1ᵃ	0.769	0.724–0.807	0.498	0.093–0.783
	2ᵇ	0.736	0.687–0.779	0.581	0.050–0.795
	3ᶜ	0.736	0.687–0.779	0.581	0.050–0.795
Female	1ᵃ	0.834	0.798–0.865	0.765	0.437–0.897
	2ᵇ	0.768	0.719–0.810	0.703	0.442–0.894
	3ᶜ	0.774	0.726–0.814	0.702	0.408–0.831

^a^Equation 1; Martín and Hernández (14); ^b^Equation 2: Elia and BAPEN (15); ^c^Equation 3: Jung et al. (16).

CI: confidence interval.

Equation 1 (14) showed the highest reliability, followed by Equation 2 (15) and Equation 3 (16).

The descriptive analysis of mean differences (measured minus estimated weight) revealed a positive bias across all equations. In men, mean differences were 13.53 ± 8.82 kg (Equation 1), 10.60 ± 10.53 kg (Equation 2), and 10.60 ± 10.53 kg (Equation 3). In women, the bias was smaller: 6.35 ± 8.55 kg, 6.62 ± 10.43 kg, and 7.06 ± 10.46 kg, respectively.

**Figure 1:**
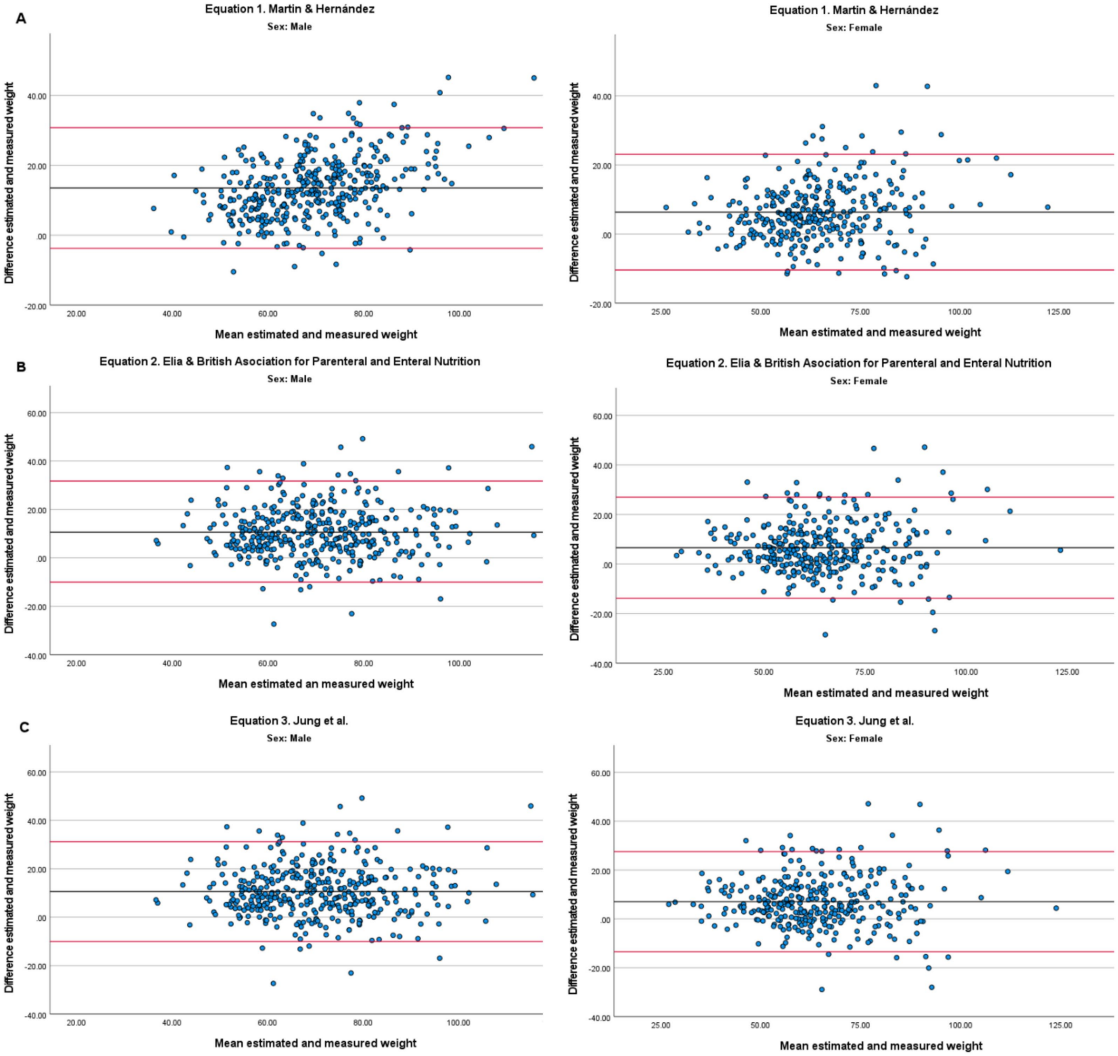
Bland–Altman diagram as a function of equation 1^A^, 2^B^ y 3^C^, disaggregated by sex. **(A)** Equation 1; Martín and Hernández (14); **(B)** Equation 2: Elia and BAPEN (15); **(C)** Equation 3: Jung et al. (16).

Figure 1 shows Bland–Altman plots for the three predictive equations (A = Equation 1, B = Equation 2, C = Equation 3), stratified by sex. The central black line represents the mean bias, and the red lines indicate the 95% limits of agreement. The dispersion of points was similar across equations, with a general tendency to underestimate measured weight, more evident in men. In women, the dispersion was narrower, reflecting greater consistency in weight estimation.

### 3.2 Development and internal validation of the new predictive equation

For model development, the total sample was randomly divided into two independent subsamples: 60% (381 men and 321 women) for model derivation and 40% (273 men and 221 women) for internal validation.

### 3.3 Model development

A multiple linear regression model was constructed for each sex, using the stepwise method, assessing at each step the increase in R-squared contributed by the variable included in the model. The dependent variable was measured weight (kg), and the potential predictors were BC, CC and KH. Model fit and standard error of the estimate are summarized in Supplementary Table S1. In both sexes, the first variable entered into the model was BC, with an R-squared of 0.517 in men and 0.602 in women. Secondly, the inclusion CC substantially improved the model fit (ΔR^2^ = 0.122 in men and ΔR^2^ = 0.103 in women). Finally, although KH was significant, its contribution resulted in only a small additional increase (ΔR^2^ = 0.023 in men and ΔR^2^ = 0.004 in women). For its balance between accuracy and simplicity, the two-variable model (BC and CC) was selected as the final predictive equation.

### 3.4 Final equations

From the unstandardized coefficients of the two-variable model, the following predictive equations were derived:

- Men: Weight (kg) = 1.755 × BC (cm) + 1.532 × CC (cm) − 26.250
- Women: Weight (kg) = 1.666 × BC (cm) + 1.239 × CC (cm) − 21.751

In both sexes, both circumferences were significant predictors (*p* < 0.001). Positive coefficients indicate a direct relationship between anthropometric measures and body weight, with BC contributing the most. Standardized coefficients (*β*), t-values, and *p*-values are detailed in Supplementary Table S2.

### 3.5 Internal validation

In the validation sample (40%), a high level of agreement was observed between the estimated and measured body weight using the two-variable model. Among men, the intraclass correlation coefficient (ICC, type A, absolute agreement, single measures) was 0.764 (95% CI: 0.710–0.810), and among women it was 0.824 (95% CI: 0.777–0.862), indicating excellent reliability and stability of the model (Table 4). Detailed ICC values for both consistency and absolute agreement are provided in Supplementary Table S3.

**Table 4:** Agreement between measured and estimated weight (two-variable model) in the validation sample.

Sex	*n*	Mean difference (kg)	SD (kg)	Absolute agreement (single measures)	95% CI
Male	273	0.24	9.37	0.764	0.710–0.809
Female	221	0.93	8.41	0.825	0.778–0.863

SD: standard deviation; CI: confidence interval.

The mean bias was minimal (0.24 ± 9.37 kg in men and 0.93 ± 8.41 kg in women), with no systematic tendency toward over- or underestimation.

**Figure 2:**
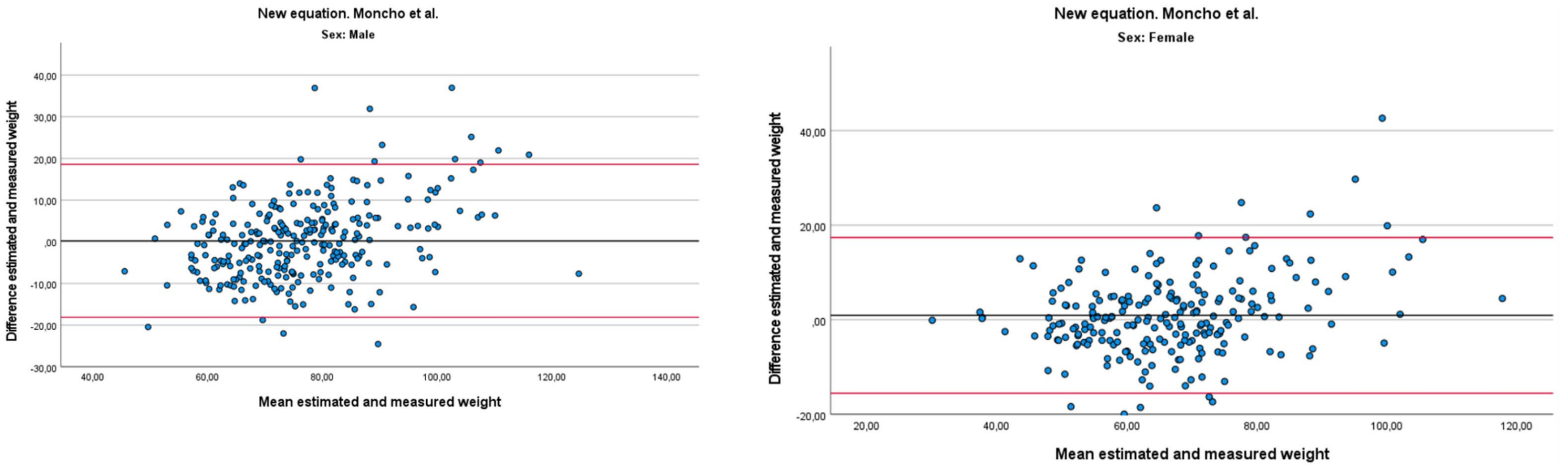
Bland–Altman plots showing the agreement between measured weight and weight estimated using the predictive equation, by sex.

Bland–Altman plots (Figure 2) confirmed the agreement between measured and estimated weight, with most points evenly distributed around the mean bias and within the 95% limits of agreement, supporting the validity and stability of the model for both sexes.

### 3.6 Impact of weight estimation on BMI and low BMI classification according to GLIM criteria

Table 5 shows BMI calculated using measured body weight and BMI derived from estimated body weight using the different equations, together with mean differences relative to measured BMI and their impact on low BMI classification according to GLIM criteria, stratified by sex.

**Table 5:** Measured and estimated body mass index (BMI), mean differences, and classification of low BMI according to GLIM criteria, by sex.

BMI	Mean	SD	Mean difference	SD	*p* value^(1)^	Low BMI (GLIM), %	*p* value^(2)^
Male							
Measured BMI	26.59	4.76	–	–	–	14.3	–
Estimated BMI – Equation 1	21.84	3.93	−4.75	3.05	<0.001	49.8	<0.001
Estimated BMI – Equation 2	22.72	4.81	−3.87	3.77	<0.001	41.7	<0.001
Estimated BMI – Equation 3	22.72	4.81	−3.87	3.77	<0.001	41.7	<0.001
Estimated BMI – Equation 4	26.55	4.21	−0.04	3.19	1.000	10.8	0.228
Female							
BMI	Mean	SD	Mean difference	SD	*p* value	Low BMI (GLIM), %	*p* value
Measured BMI	27.07	5.64	–	–	–	15.7	–
Estimated BMI – Equation 1	24.01	5.38	−3.07	3.12	<0.001	32.6	<0.001
Estimated BMI – Equation 2	23.90	6.04	−3.17	3.89	<0.001	36.5	<0.001
Estimated BMI – Equation 3	23.70	6.22	−3.38	3.88	<0.001	39.9	<0.001
Estimated BMI – Equation 4	26.65	5.07	−0.42	3.20	0.324	16.3	0.800

BMI: body mass index; SD: standard deviation; GLIM: Global Leadership Initiative on Malnutrition. ^(1)^ Paired t-test compare mean BMI values Bonferroni correction applied for multiple comparison; ^(2)^ McNemar’s test was used to analyse differences in low BMI classification according to GLIM criteria Bonferroni correction applied for multiple comparison.

Among men, previously published equations showed a significant underestimation of BMI, with mean differences ranging from −3.87 to −4.75 kg/m^2^ (all *p* < 0.001). This underestimation resulted in a substantial increase in the proportion of patients classified as having low BMI, rising from 14.3% when measured BMI was used to 41.7–49.8% when BMI was calculated using Equations 1 to 3 (*p* < 0.001). In contrast, the new equation (Equation 4) showed a minimal and non-significant mean difference compared with measured BMI (−0.04 ± 3.19 kg/m^2^; *p* = 1.000), with a proportion of low BMI similar to that observed using measured weight (10.8%; *p* = 0.228).

Among women, Equations 1 to 3 also significantly underestimated BMI, with mean differences ranging from −3.07 to −3.38 kg/m^2^ (*p* < 0.001), increasing the prevalence of low BMI from 15.7% to 32.6–39.9% (*p* < 0.001). Similarly, to men, Equation 4 showed a non-significant difference compared with measured BMI (−0.42 ± 3.20 kg/m^2^; *p* = 0.324) and did not result in relevant changes in low BMI classification according to GLIM criteria (16.3%; *p* = 0.800).

## 4 Discussion

The results obtained in this study show wide variability in measured body weight (range: 30–138 kg) in hospitalised patients aged 65 years or older, reflecting the anthropometric heterogeneity of this population and highlighting the need for valid tools for estimating weight when direct measurement is not possible.

The three predictive equations analysed (14–16) showed a systematic underestimation of measured weight, which was particularly marked in men. In addition, it was observed that the higher the body weight, the greater the discrepancy between the estimated and measured weight, particularly with Equation 1 (14). This pattern is clinically relevant, as underestimating weight can lead to errors in drug dosing, inaccurate BMI estimates, and biased assessments of nutritional status (19).

In terms of reliability, Equation 1 (14) showed the highest ICC values for consistency (≈ 0.77 in men and ≈ 0.83 in women), but with lower absolute agreement in men (≈ 0.50) than in women (≈ 0.76). Equations 2 (15) and 3 (16) presented similar results between sexes, which was to be expected as they shared the same formula for men. From a methodological point of view, the ICC of absolute agreement is the most appropriate indicator for assessing clinical accuracy, as it captures both proportional and constant errors, while the ICC of consistency is useful for identifying possible calibration adjustments in the equation. Overall, the values obtained suggest moderate agreement in women and lower agreement in men, highlighting the need to optimize these formulas for elderly hospitalised patients.

Comparing our results with those originally reported by Martín & Hernández (14), we observed a lower model fit (R^2^ = 0.65 in men and 0.69 in women versus 0.85 and 0.88, respectively, in the original study). These differences can be explained by population disparity (Venezuelans aged 20–50 years versus Spanish patients ≥ 65 years), functional status (general population vs. hospitalised population) and clinical context (reduced mobility). This confirms the low transferability of anthropometric equations between settings and age groups, reinforcing the need to develop specific formulas for hospitalised geriatric patients.

In this regard, the study by Cattermole et al. (20) showed that BC can be used to estimate weight in adults and adolescents with high concordance (≥ 60% of estimates within ±10% of actual weight), using data derived from the National Health and Nutrition Examination Survey (NHANES). Although their results support the potential of methods based on simple measurements, this work was carried out in the general population and not in elderly hospitalised patients, which again highlights the importance of validating and adapting these approaches to clinical contexts with limited mobility.

Guerra et al. (8) developed and validated specific equations for older adults that incorporated five anthropometric variables (height, brachial circumference, calf circumference, waist circumference, and triceps skinfold thickness), achieving R^2^ > 0.80 and standard errors ≤ 5.5 kg (21). Although these multivariate strategies improve accuracy, their clinical application may be limited by the complexity of the measurements and the availability of specific equipment. Consistently, Silva et al. (22) observed that the concordance between different methods of estimating weight and height in older people depends heavily on the type of anthropometric measurement and the clinical context, highlighting the need to validate equations in the populations where they are to be applied.

In this current study, the final two-variable model (BC + CC) showed an optimal balance between accuracy and clinical applicability, with significant increases in R^2^ when incorporating the second variable (ΔR^2^ = 0.122 in men and 0.103 in women) and a minimal mean bias (< 1 kg). In the validation sample, the model achieved high concordance with measured weight (ICC = 0.764 in men and 0.824 in women), with no systematic tendency to over- or underestimate in the Bland–Altman plots. These results confirm that an equation based solely on BC and CC can be a reliable, reproducible, and feasible tool in the hospital setting for people aged 65 years or older.

The results of this study indicate that discrepancies between measured and estimated body weight are not only statistically relevant but also have direct clinical implications through their impact on BMI and low BMI classification according to GLIM criteria. In this study, previously published equations systematically underestimated BMI, resulting in a substantial increase in the proportion of patients classified as having low BMI, particularly among men. This finding represents a clinically relevant issue, as it may lead to an overestimation of the prevalence of low BMI in hospitalised older adults when estimated weight is used. These findings are consistent with previous evidence indicating that imprecision in weight-dependent parameters may translate into clinically relevant downstream effects, as variability in calculated energy requirements has been reported depending on the assessment method used (9). In contrast, the newly developed equation showed minimal and non-significant differences compared with BMI calculated using measured weight, maintaining a proportion of low BMI similar to that observed with direct measurement. These findings reinforce the importance of having weight estimation tools specifically adapted to the hospitalised geriatric population, not only to improve anthropometric accuracy but also to reduce the risk of clinical interpretations based on inaccurate BMI estimation.

### 4.1 Implications for clinical practice

The results of this study have direct implications for hospital practice. Firstly, the new equation developed, based solely on brachial circumference BC and CC, facilitates rapid and reliable estimation of body weight in elderly hospitalised patients with reduced mobility, in whom direct measurement is often unfeasible. Its clinical applicability is high, as both measurements can be taken with basic materials (non-extendable tape measure) and by healthcare personnel with minimal training, following a standardized protocol.

This tool can be easily integrated into systematic nutritional assessment, supporting the early identification of malnutrition, BMI estimation, and the adjustment of body weight-dependent drug doses. Furthermore, its use can optimise nutritional and therapeutic care, reduce errors arising from subjective weight estimation, and improve clinical safety.

The proposed model aligns with international recommendations on early, personalized nutritional screening in hospitalised elderly patients (23), promoting evidence-based practice focused on operational feasibility.

Finally, the equation could serve as a basis for the development of digital tools or automated clinical calculators that can be integrated into electronic health records, facilitating their routine implementation and dissemination at different levels of care.

### 4.2 Methodological limitations and considerations

This study has some limitations inherent to the development and internal validation of predictive equations. Firstly, the validation was internal, so although the results show high concordance and minimal mean bias, external validation in other cohorts and hospital settings will be necessary before recommending its widespread use. Given that the model was developed in patients whose body weight could be directly measured, its generalizability to patients in whom weight assessment is not feasible may be limited. However, data were collected from multiple hospital units and different hospitals, increasing sample heterogeneity, which may partially mitigate this limitation. Secondly, although the final model explained a considerable proportion of the variability in body weight, additional variables such as height, oedema, or body composition were not included. This decision was intentional, as the main objective of the study was to develop a simple, rapid equation that could be applied in routine clinical practice, especially in hospitalised patients with reduced mobility. Including more variables could marginally increase accuracy, but at the cost of reducing its feasibility and reproducibility in real hospitalisation conditions.

Furthermore, the sample was obtained consecutively from nine public hospitals, which could limit its representativeness of other clinical settings (e.g., primary care or social-health centres). However, the multicentre design and the homogeneity of the data collection procedures (ensured through specific training, audiovisual material and centralised supervision within the NUTRIFAG project) confer high consistency and reliability on the anthropometric measurements taken.

Although the use of the stepwise method may overfit the resulting model, in the present study, stepwise regression was used as an initial procedure, with the aim of obtaining the model with the highest R-squared, but it was not the only criterion for its specification. It should be noted that the procedure did not exclude any of the candidate variables, as all predictors initially included remained statistically significant in the final model. Consequently, the resulting model is equivalent to a fully specified model that incorporates all covariates considered relevant from a theoretical and clinical perspective.

Overall, these limitations do not invalidate the findings, but rather delimit their applicability and reinforce the need for external validation of the equation in diverse populations and settings to confirm its clinical utility.

## 5 Conclusion

This study developed and internally validated a new equation for estimating body weight based on two simple anthropometric measurements (BC and CC), which showed high concordance and minimal bias with respect to measured weight. In addition, it provided a BMI estimation closer to that obtained from measured body weight, compared with previously published equations. Due to its accuracy, simplicity, and operational feasibility, this equation represents a useful clinical tool for estimating weight in hospitalised older adults with reduced mobility, contributing to a safer nutritional and therapeutic assessment. Future studies should conduct external validation in different healthcare settings and assess its impact on routine clinical practice.

## NUTRIFAG Group

The NUTRIFAG Working Group that has collaborated in: Ángel Luís Abad-González, Marco Aldonza-Torres, Elena Altarribas-Bolsa, Rahma Amrani, María Argente-Pla, Esther Barrufet-Alcántara, Joan Blanco-Blanco, Rafaela Camacho-Bejarano, María Ángeles Canovas Molina, Francisco Javier Carrasco-Sánchez, Cristina Carretero-Randez, Pedro Raúl Castellano-Santana, Jenifer Castellano-Santana, María Trinidad Castillo-García, Sandra Cebrián-Jimeno, Marta Charlo-Bernardos, Esperanza Ciérvide-Górriz, Rosa Ana Clement-Santamaría, María José Compañ-Aguilar, Clara De la Fuente-Gómez, Manuela Domingo-Pozo, Cristina Domínguez-Gadea, Yesmina El-Khattabi-Ofkir, Purificación Enguix-Bou, Marina Figueras-Acebillo, Ascensión Franco-Bernal, José Ángel Franco-Romero, Agnès Gabarra-Mesalles, Miguel Galeote-Muñoz, Carla García Martín, María Amparo Garrigues-Esteve, Antonia Gomariz-Martínez, María Bienvenida Gómez-Sánchez, Víctor Manuel González-Chordá, María del Pino Hernández-Plata, Beatriz Herrero-Cortina, Cristina Hurtado-Soler, Érika María Lorenzo-Ramos, Anna Marco-Mitjavila, Amando Márquez-Sixto, Idaira Martín-Santana, Laura Meseguer-Galiana, Joaquín Moncho, Mercedes del Pilar Montalván-Pelegrín, Jesica Montero-Marco, Mª José Morano-Torrescusa, Patricia Navarro-Llopes, Mª Julia Ocón-Bretón, María Isabel Orts-Cortés (PI), Cristina Pérez-Bello, Laura Pérez-Navarro, Paloma Portillo-Ortega, Vanesa Ramos-Abril, Isabel Rebollo-Pérez, Beatriz, Rodríguez-Ojeda, Margarita Rodríguez-Pérez, Aránzazu Ruiz-Heras-Hera, Lourdes Salinero-González, Ana Belén Sánchez-García, Mª Dolores Santos-Rey, Mónica Timoneda-Company, Eva María Trescastro-López, Erika Lexandra Vieira-Maroun, Fabiola Zambom-Ferraresi, Antonia Inmaculada Zomeño-Ros, Ginesa Zomeño-Ros.

## Data Availability

The raw data supporting the conclusions of this article will be made available by the authors, without undue reservation.
