# Supplementary material for: Weight estimation in people aged 65 years and over admitted to hospitalisations units
Source: Front Nutr. 2026 Mar 4;13:1756595. doi: 10.3389/fnut.2026.1756595 (PMC12998235; doi:10.3389/fnut.2026.1756595)
Supplement: Supplementary file 1 [file Table_1.docx]

Supplementary Material

# Supplementary Data

**Description of anthropometric variables and measuring instruments**

Descriptive legend: In this supplementary material file, the anthropometric variables used in the study will be described and the instruments and method of performing the body measurements used in the study will be explained in detail.

*Anthropometric Variables*

Weight (kg) and proxy measures for estimating weight (depending on the method used) were obtained: mid-arm circumference (BC), mid-calf circumference (CC), and heel-knee height (HK). The procedure for measuring these variables and the instruments used are described below:

1. *Measured weight* (1-3)

This is the measurement of body mass expressed in kilograms collected during the first 24-48 hours of the patient's admission.

The SECA Clara 803 digital scale (white) with a capacity of up to 150 kg, which has a division of 100 g and a weight of 1.5 kg was used. It is battery-operated and its dimensions (WxHxD) are 316 x 37 x 326 mm. The scale was placed on an accessible flat horizontal surface in each of the participating rooms. To obtain the measurement, the subject was positioned on the scale with feet parallel in the center and without shoes, upright, looking straight ahead, and with arms falling naturally to the sides.

*B. Estimated weight* (1-3)

*B.1. Mid-Arm circumference (BC)*

Technique:

1. Bend the arm until it forms a 90º angle with the forearm.

2. With the aid of the tape measure, calculate and mark the midpoint between the distance between the acromion of the shoulder and the olecranon of the elbow.

3. Extend the arm along the body and encircle the marked point with the tape measure, without compressing it. In patients with limited mobility, the left arm should be held extended along the body with the palm facing upwards.

5. Take three measurements at a time and calculate the mean.

*B.2. Mid-Calf Circumference (CC*)

Technique:

1. The patient should be seated with both legs apart, with the bent leg forming a right angle between heel and knee; if the measurement is taken with the subject standing, weight should be distributed evenly over both feet. Whenever possible, the measurement should be taken on the non-dominant leg.

2. The assessment is made by standing on the patient's right side, facing the lateral side of the calf, and keeping the tape perpendicular to the calf axis.

3. Ideally, three measurements are taken, noting the value measured at its most protruding point.

Technique if the patient has total limitation of mobility:

1. The patient remains supine with the non-dominant leg knee flexed at a 90º angle.

2. The contour of the widest part of the calf is measured with the tape measure, without compressing the tissues.

3. Three measurements are taken, noting the value measured at its most protruding point.

*B.3. Heel-knee height (KH)*

Technique:

1. Sit the patient in a chair, barefoot, and instruct him/her to bend the knee at a right angle.

2. Hold the tape measure between the third and fourth fingers, so that the zero is just below the fingers.

3. Rest your hand flat on the subject's thigh, about 4 cm behind the knee.

4. Extend the tape measure down the side of the leg, passing through the bony protrusion of the ankle (lateral malleolus), to the base of the heel. Measure the distance, rounding off to 0.5 cm.

5. Finally, record the length obtained.

Technique if the patient has total limitation of mobility:

1. The patient may be seated or lying down, provided the knee is flexed at a 90° angle.

2. The tip of the tape measure is placed under the heel and the tape is extended to the top of the kneecap.

3. Three measurements are taken, noting the measured value for each measurement.

*C. Measured height*

Height was measured using a wall-mounted stadiometer (SECA 206, SECA GmbH & Co. KG, Hamburg, Germany) in each participating unit. Participants were asked to remove their shoes and stand upright with both feet flat on the floor and heels against the stadiometer (or the wall). The participant’s head position was checked to ensure they were looking straight ahead. The headpiece was then lowered until it gently touched the top of the head. Height was recorded to the nearest measurement unit indicated by the device.

References

Todorovic V, Russell C, Elia M, editores. Manual explicativo ‘MUST’: Guía para el ‘Instrumento universal para el cribado de la malnutrición’ (‘MUST’) para adultos. 2ª ed. Redditch: British Association for Parenteral and Enteral Nutrition (BAPEN); 2011.

Elia M, British Association for Parenteral and Enteral Nutrition. Guidelines for detection and management of malnutrition. Maidenhead: BAPEN; 2000

Anthony PS. Nutrition screening tools for hospitalized patients. Nutr Clin Pract. 2008 Aug 5; 23(4): 373–82. doi: 10.1177/0884533608321130

# Supplementary Figures and Tables

## Supplementary Tables

**Supplementary Table S1. Multiple linear regression models for body weight estimation using anthropometric predictors, by sex**

| **Sex** | **Model** | **Variables included** | **R** | **R²** | **Adjusted R²** | **Standard error (kg)** | Δ**R²** | **F change** | **df1** | **df2** | **p** |
| --- | --- | --- | --- | --- | --- | --- | --- | --- | --- | --- | --- |
| **Male (n = 381)** | 1^a^ | BC | 0.719 | 0.517 | 0.516 | 10.18 | 0.517 | 405.72 | 1 | 379 | < 0.001 |
|  | 2^b^ | BC + CC | 0.800 | 0.639 | 0.637 | 8.81 | 0.122 | 128.12 | 1 | 378 | < 0.001 |
|  | 3^c^ | BC + CC + KH | 0.814 | 0.663 | 0.660 | 8.53 | 0.023 | 26.14 | 1 | 377 | < 0.001 |
| **Female (n = 321)** | 1^a^ | BC | 0.776 | 0.602 | 0.600 | 9.87 | 0.602 | 481.66 | 1 | 319 | < 0.001 |
|  | 2^b^ | BC + CC | 0.839 | 0.704 | 0.703 | 8.52 | 0.103 | 110.75 | 1 | 318 | < 0.001 |
|  | 3^c^ | BC + CC + KH | 0.842 | 0.709 | 0.706 | 8.47 | 0.004 | 4.67 | 1 | 317 | 0.032 |

**a. Predictors: (Constant), Mid-arm circumference (BC) (auto-calculated).**

**b. Predictors: (Constant), Mid-arm circumference (BC) (auto-calculated), Mid-Calf circumference (CC) (average of three measurements) in cm (auto-calculated).**

**c. Predictors: (Constant), Mid-arm circumference (BC) (auto-calculated), Mid-Calf circumference (CC) (average of three measurements) in cm (auto-calculated), Heel–knee length (KH) (cm).**

**d. Dependent variable: Measured weight (MW) (kg).
Abbreviations: BC = mid-arm circumference; CC = mid-calf circumference; KH = heel–knee length; AW = admission weight.**

Supplementary Table S2. Coefficients of multiple linear regression models for body weight estimation (kg)

| Sex | Model | Variable | B | Standard error | Beta | t | p |
| --- | --- | --- | --- | --- | --- | --- | --- |
| **Male** | 1 | (Constant) | 0.076 | 3.803 | — | 0.020 | 0.984 |
|  |  | BC (cm) | 2.696 | 0.134 | 0.719 | 20.143 | <0.001 |
|  | 2 | (Constant) | −26.250 | 4.030 | — | −6.514 | <0.001 |
|  |  | BC (cm) | 1.755 | 0.143 | 0.468 | 12.308 | <0.001 |
|  |  | CC (cm) | 1.532 | 0.135 | 0.430 | 11.319 | <0.001 |
|  | 3 | (Constant) | −44.196 | 5.249 | — | −8.421 | <0.001 |
|  |  | BC (cm) | 1.643 | 0.140 | 0.438 | 11.754 | <0.001 |
|  |  | CC (cm) | 1.469 | 0.132 | 0.413 | 11.165 | <0.001 |
|  |  | KH (cm) | 0.467 | 0.091 | 0.159 | 5.113 | <0.001 |
| **Female** | 1 | (Constant) | −0.258 | 3.153 | — | −0.082 | 0.935 |
|  |  | BC (cm) | 2.370 | 0.108 | 0.776 | 21.947 | <0.001 |
|  | 2 | (Constant) | −21.751 | 3.401 | — | −6.396 | <0.001 |
|  |  | BC (cm) | 1.666 | 0.115 | 0.545 | 14.530 | <0.001 |
|  |  | CC (cm) | 1.239 | 0.118 | 0.395 | 10.524 | <0.001 |
|  | 3 | (Constant) | −30.507 | 5.279 | — | −5.779 | <0.001 |
|  |  | BC (cm) | 1.675 | 0.114 | 0.548 | 14.680 | <0.001 |
|  |  | CC (cm) | 1.196 | 0.119 | 0.381 | 10.073 | <0.001 |
|  |  | KH (cm) | 0.218 | 0.101 | 0.067 | 2.160 | 0.032 |

**Dependent variable: Measured weight (kg).**

**Abbreviations: BC = mid-arm circumference; CC = mid-calf circumference; KH = heel–knee length.**

Supplementary Table S3. Intraclass correlation coefficients for the final predictive equation (Moncho et al.)

| Sex | Predictive equation | Consistency (single measures) | 95% CI | Absolute agreement (single measures) | 95% CI |
| --- | --- | --- | --- | --- | --- |
| **Male** | New equation (BC + CC) | 0.764 | 0.710–0.809 | 0.764 | 0.710–0.810 |
| Female | New equation (BC + CC) | 0.825 | 0.778–0.863 | 0.824 | 0.777–0.862 |

**BC = mid-arm circumference; CC = mid-calf circumference**
